# Supplementary material for: Membrane transporter dimerization driven by differential lipid solvation energetics of dissociated and associated states
Source: eLife. 2021 Apr 7;10:e63288. doi: 10.7554/eLife.63288 (PMC8116059; doi:10.7554/eLife.63288)
Supplement: Figure 2—source data 1. — A total of 0.7 ms total simulation time was carried out for this study. [file elife-63288-fig2-data1.docx]

**Figure 2 – source data 1. CGMD simulation specifications.** A total of 0.7 ms total simulation time was carried out for this study.

|  | **Dimer** | **Monomer** | | | | | **Pure Membrane** | | | |
| --- | --- | --- | --- | --- | --- | --- | --- | --- | --- | --- |
| **Component** | **100% PO** | **100% PO** | **99% PO**  **1% DL** | **90% PO**  **10% DL** | **70% PO**  **30% DL** | **50% PO**  **50% DL** | **100% PO** | **90% PO 10% DL** | **70% PO 30% DL** | **50% PO 50% DL** |
| Protein | 2 | 1 | 1 | 1 | 1 | 1 | 0 | 0 | 0 | 0 |
| POPE | 1814 | 1819 | 1954 | 1672 | 1251 | 839 | 892 | 802 | 616 | 498 |
| POPG | 959 | 965 | 977 | 823 | 626 | 425 | 296 | 402 | 320 | 222 |
| DLPE | 0 | 0 | 19 | 214 | 629 | 976 | 0 | 88 | 268 | 448 |
| DLPG | 0 | 0 | 11 | 109 | 343 | 479 | 0 | 46 | 104 | 220 |
| Na^+^ | 1898 | 1710 | 1377 | 1696 | 1730 | 1514 | 572 | 576 | 554 | 582 |
| Cl^–^ | 953 | 748 | 390 | 771 | 764 | 613 | 126 | 128 | 130 | 140 |
| H_2_O | 54,956 | 35,382 | 33,150 | 36,333 | 37,450 | 26,814 | 10,112 | 10,304 | 10,444 | 11,179 |
|  |  |  |  |  |  |  |  |  |  |  |
| System size (nm) | 29.4 x  29.4 x 12.0 | 29.1 x 29.1 x 9.4 | 30.4 x 30.4 x 8.6 | 29.0 x 29.0 x 9.5 | 29.0 x 29.0 x 9.6 | 27.9 x 27.9 x 8.3 | 19.9 x 19.9 x 7.4 | 19.8 x 19.8 x 7.4 | 19.5 x 19.5 x 7.5 | 19.5 x 19.5 x 7.6 |
| Number of replicas | 10 | 8 | 8 | 8 | 8 | 8 | 8 | 8 | 8 | 8 |
| Simulation time per replica (μs) | 9.6 - 12.9 | 7.4 | 20.0 | 6.1 | 6.0 | 7.8 | 6.0 | 6.7 | 7.8 | 6.8 |
| Total simulation time (μs) | 107.4 | 59.1 | 160.0 | 55.0 | 48.3 | 55.0 | 48.0 | 53.6 | 62.7 | 54.2 |
|  |  |  |  |  |  |  |  |  |  |  |
