# Supplementary material for: Membrane transporter dimerization driven by differential lipid solvation energetics of dissociated and associated states
Source: eLife. 2021 Apr 7;10:e63288. doi: 10.7554/eLife.63288 (PMC8116059; doi:10.7554/eLife.63288)
Supplement: Figure 3—source data 1. — Measurement conducted on liposomes post freeze-thaw in the multi-lamellar state. Data represented as mean ± sem. Statistical analysis carried out using a two-tailed unpaired parametric student's t-test compared to the 0% DL samples. [file elife-63288-fig3-data1.docx]

**Figure 3 - source data 1. Differential scanning calorimetry for mixed DL/PO 2:1 PE/PG membranes**. Measurement conducted on liposomes post freeze-thaw in the multi-lamellar state. Data represented as mean ± sem. Statistical analysis carried out using a two-tailed unpaired parametric student's t-test compared to the 0% DL samples.

| **DL (%)** | **T_m_ (°C)** | **n** | ***P*-value** |
| --- | --- | --- | --- |
| 0 | 20.3 ± 0.2 | 4 |  |
| 10 | 18.5 ± 0.4 | 3 | **, 0.0046 |
| 30 | 14.6 ± 0.8 | 3 | ***, 0.0005 |
| 50 | 16.1 ± 0.7 | 3 | **, 0.0014 |
| 70 | 19.1 ± 0.4 | 3 | *, 0.0333 |
| 100 | 25.9 ± 0.7 | 3 | ***, 0.0004 |
