# Supplementary material for: Membrane transporter dimerization driven by differential lipid solvation energetics of dissociated and associated states
Source: eLife. 2021 Apr 7;10:e63288. doi: 10.7554/eLife.63288 (PMC8116059; doi:10.7554/eLife.63288)
Supplement: Figure 3—source data 2. — 100 nm extruded vesicles, at 25°C. Data represent best-fit ± standard error. A description of the CGMD analysis is in Figure 2—figure supplement 2. [file elife-63288-fig3-data2.docx]

**Figure 3 – source data 2. SANS bilayer thickness analysis for mixed DL/PO 2:1 PE/PG membranes**. 100 nm extruded vesicles, at 25°C. Data represent best-fit ± standard error. A description of the CGMD analysis is in **Fig. 2 – fig. supp. 2.**

| **SANS data** | | | | **CGMD analysis** | |
| --- | --- | --- | --- | --- | --- |
| **2:1 PE/PG** | **d_b_ (nm)** | **d_w_ (nm)** | **% ULV** | **d_esters_ (nm)** | **d_PO4_ (nm)** |
| 0% DL | 3.70 ± 0.02 | 6.50 ± 0.03 | 85 | 3.20 | 4.06 |
| 10% DL | 3.55 ± 0.01 | 5.85 ± 0.03 | 85 | 3.15 | 4.02 |
| 30% DL | 3.46 ± 0.02 | 6.11 ± 0.02 | 85 | 3.06 | 3.92 |
| 50% DL | 3.21 ± 0.01 | 6.49 ± 0.03 | 87 | 2.95 | 3.81 |
| 70% DL | 3.08 ± 0.02 | 6.64 ± 0.03 | 90 | 2.85 | 3.72 |
| 90% DL | 2.98 ± 0.02 | 6.65 ± 0.04 | 90 | 2.39 | 3.66 |
| 100% DL | 2.99 ± 0.02 | 6.73 ± 0.03 | 95 |  |  |
| **PC** | **(Kučerka et al., 2011)** |  |  |  |  |
| 0% DL | 3.98 ± 0.08 |  |  |  |  |
| 100% DL | 3.30 ± 0.07 |  |  |  |  |
| **PG**** | **(Pan et al., 2014)** |  |  |  |  |
| 0% DL | 3.85 |  |  |  |  |
| 100% DL | 3.14 |  |  |  |  |
