# Supplementary material for: Membrane transporter dimerization driven by differential lipid solvation energetics of dissociated and associated states
Source: eLife. 2021 Apr 7;10:e63288. doi: 10.7554/eLife.63288 (PMC8116059; doi:10.7554/eLife.63288)
Supplement: Figure 4—source data 1. — From single preparation of 400 nm extruded vesicles (n = 1). [file elife-63288-fig4-data1.docx]

**Figure 4 - source data 1. Cryo-EM radii of 20% DL 2:1 POPE/POPG**. From single preparation of 400 nm extruded vesicles (n = 1).

| **r, nm** | **P_radius_** | **F_SA_** | **Cumulative F_SA_** |
| --- | --- | --- | --- |
| 2.5 | 0 | 0 | 0 |
| 7.5 | 0.0129 | 0.00018 | 0.00018 |
| 12.5 | 0.0516 | 0.00201 | 0.00219 |
| 17.5 | 0.1226 | 0.00936 | 0.01155 |
| 22.5 | 0.0387 | 0.00489 | 0.01644 |
| 27.5 | 0.0839 | 0.01582 | 0.03226 |
| 32.5 | 0.0516 | 0.01360 | 0.04586 |
| 37.5 | 0.0839 | 0.02941 | 0.07527 |
| 42.5 | 0.07097 | 0.03197 | 0.10724 |
| 47.5 | 0.05807 | 0.03267 | 0.13991 |
| 52.5 | 0.10323 | 0.07096 | 0.21087 |
| 57.5 | 0.03871 | 0.03192 | 0.24278 |
| 62.5 | 0.04516 | 0.04400 | 0.28678 |
| 67.5 | 0.05807 | 0.06598 | 0.35276 |
| 72.5 | 0.03226 | 0.04229 | 0.39504 |
| 77.5 | 0.00645 | 0.00967 | 0.40470 |
| 82.5 | 0.01290 | 0.02190 | 0.42661 |
| 87.5 | 0.00645 | 0.01232 | 0.43893 |
| 92.5 | 0.01290 | 0.02753 | 0.46646 |
| 97.5 | 0.00645 | 0.01530 | 0.48175 |
| 102.5 | 0.01290 | 0.03381 | 0.51556 |
| 107.5 | 0.00645 | 0.01859 | 0.53416 |
| 112.5 | 0.00645 | 0.02036 | 0.55452 |
| 117.5 | 0 | 0 | 0.55452 |
| 122.5 | 0.00645 | 0.02415 | 0.57866 |
| 127.5 | 0.00645 | 0.02616 | 0.60482 |
| 132.5 | 0 | 0 | 0.60482 |
| 137.5 | 0.01290 | 0.06084 | 0.66566 |
| 142.5 | 0.00645 | 0.03267 | 0.69833 |
| 147.5 | 0.01936 | 0.10502 | 0.80335 |
| 152.5 | 0.00645 | 0.03742 | 0.84076 |
| 157.5 | 0.00645 | 0.03991 | 0.88068 |
| 162.5 | 0 | 0 | 0.88068 |
| 167.5 | 0 | 0 | 0.88068 |
| 172.5 | 0 | 0 | 0.88068 |
| 177.5 | 0 | 0 | 0.88068 |
| 182.5 | 0 | 0 | 0.88068 |
| 187.5 | 0.00645 | 0.05657 | 0.93724 |
| 192.5 | 0 | 0 | 0.93724 |
| 197.5 | 0.00645 | 0.06276 | 1 |
