# Supplementary material for: Membrane transporter dimerization driven by differential lipid solvation energetics of dissociated and associated states
Source: eLife. 2021 Apr 7;10:e63288. doi: 10.7554/eLife.63288 (PMC8116059; doi:10.7554/eLife.63288)
Supplement: Figure 4—source data 2. — Data represent mean ± sem, n independent protein purifications and reconstitutions, with each sample measured as the average of >3 replicate measurements. Statistical analysis was calculated using a two-tailed unpaired parametric student t-test compared to the 0% DL data set (*p<0.05; **p<0.01; ***p<0.001). [file elife-63288-fig4-data2.docx]

**Figure 4 - source data 2. Chloride transport of CLC-ec1-Cy5 in DL/PO proteoliposomes**. Data represent mean ± sem, n independent protein purifications and reconstitutions, with each sample measured as the average of > 3 replicate measurements. Statistical analysis was calculated using a two-tailed unpaired parametric student t-test compared to the 0% DL data set (*, *P* < .05; **, *P* < .01; ***, *P* < .001).

| **DL (%)** | **incubation time (d)** | **P_Cy5_** | **F_0,vol._** | ***P*-value** | **k_P_ (norm. Cl^-^/s)** | ***P*-value** | **k_init._ (norm. Cl^-^/s)** | ***P*-value** | **n** |
| --- | --- | --- | --- | --- | --- | --- | --- | --- | --- |
| 0 | 7.3 ± 4.8 | 0.69 ± 0.01 | 0.25 ± 0.02 |  | 0.026 ± 0.001 |  | 0.020 ± 0.002 |  | 3 |
| 1 | 4.0 ± 2.0 | 0.66 ± 0.02 | 0.25 ± 0.01 | ns, 0.98 | 0.025 ± 0.001 | ns, 0.81 | 0.020 ± 0.001 | ns, 0.89 | 4 |
| 10 | 7.0 ± 3.4 | 0.68 ± 0.01 | 0.26 ± 0.02 | ns, 0.92 | 0.025 ± 0.005 | ns, 0.92 | 0.017 ± 0.002 | ns, 0.27 | 4 |
| 20 | 6.4 ± 2.7 | 0.67 ± 0.01 | 0.33 ± 0.08 | ns, 0.50 | 0.015 ± 0.002 | **, 0.008 | 0.010 ± 0.002 | *, 0.02 | 5 |
| 40 | 7.0 ± 3.3 | 0.67 ± 0.01 | 0.57 ± 0.10 | *, 0.046 | 0.010 ± 0.001 | ***, 0.0001 | 0.005 ± 0.001 | ***, 0.0008 | 4 |
