# Supplementary material for: Membrane transporter dimerization driven by differential lipid solvation energetics of dissociated and associated states
Source: eLife. 2021 Apr 7;10:e63288. doi: 10.7554/eLife.63288 (PMC8116059; doi:10.7554/eLife.63288)
Supplement: Figure 4—source data 3. — χ* is the reactive molar ratio of protein subunits and lipids, based on the observed mole fraction calculated from the protein and lipid quantification assays, and assuming the reaction occurs between oriented species in the membrane, χ* = χ/2. Data are represented as mean ± standard error. Sample numbers, n, are listed separately for (mole fraction quantification, photobleaching analysis). [file elife-63288-fig4-data3.docx]

**Figure 4 - source data 3. Photobleaching data for monomeric control CLC-ec1 I201W/I422W, WW-Cy5 in 20% DL, 80% PO, 2:1 PE/PG lipids.** 𝜒* is the reactive molar ratio of protein subunits and lipids, based on the observed mole fraction calculated from the protein and lipid quantification assays, and assuming the reaction occurs between oriented species in the membrane, 𝜒* = 𝜒/2. Data are represented as mean ± standard error. Sample numbers, n, are listed separately for (mole fraction quantification, photobleaching analysis).

| **density (μg/mg)** | **𝜒* (subunits/lipid)** | **P_Cy5_** | **incubation time (d)** | **P_1_** | **P_2_** | **P_3+_** | **n** |
| --- | --- | --- | --- | --- | --- | --- | --- |
| 0.0001 | (8.24 ± 3.07) x 10^-10^ | 0.78 ± 0.03 | 4.0 ± 0.0 | 0.74 ± 0.11 | 0.21 ± 0.07 | 0.06 ± 0.04 | (2,2) |
| 0.001 | (3.39 ± 0.57) x 10^-9^ | 0.77 ± 0.02 | 3.7 ± 0.3 | 0.76 ± 0.04 | 0.20 ± 0.03 | 0.04 ± 0.00 | (2,3) |
| 0.01 | (2.92 ± 0.21) x 10^-8^ | 0.77 ± 0.02 | 3.7 ± 0.3 | 0.79 ± 0.01 | 0.17 ± 0.01 | 0.04 ± 0.01 | (2,3) |
| 0.1 | (2.77 ± 0.31) x 10^-7^ | 0.77 ± 0.02 | 3.7 ± 0.3 | 0.63 ± 0.04 | 0.24 ± 0.02 | 0.13 ± 0.03 | (2,3) |
| 0.5 | (2.05 ± 0.42) x 10^-6^ | 0.78 ± 0.03 | 4.0 ± 0.0 | 0.38 ± 0.04 | 0.23 ± 0.01 | 0.41 ± 0.03 | (2,2) |
