# Supplementary material for: Membrane transporter dimerization driven by differential lipid solvation energetics of dissociated and associated states
Source: eLife. 2021 Apr 7;10:e63288. doi: 10.7554/eLife.63288 (PMC8116059; doi:10.7554/eLife.63288)
Supplement: Figure 4—source data 6. — CLC-ec1-Cy5 proteoliposomes (0.2 μg/mg) in 100% PO, 2:1 PE/PG were either (A) unmodified, (B) fused with 100% PO 2:1 PE/PG liposomes or (C) fused with 40% DL, 60% PO 2:1 PE/PG liposomes by multiple freeze-thaw cycles. Data represented as mean ± sem, n = 3 independent samples. p-Values are calculated using a two-tailed student's t-test on the P1 photobleaching data, and using the χ2 test on the (P1,P2,P3+) photobleaching probability distributions, designated in brackets (*p<0.05; **p<0.01). [file elife-63288-fig4-data6.docx]

**Figure 4 - source data 6. Shift in dimer equilibrium upon fusion with DL containing vesicles.** CLC-ec1-Cy5 proteoliposomes (0.2 μg/mg) in 100% PO, 2:1 PE/PG were either (A) unmodified, (B) fused with 100% PO 2:1 PE/PG liposomes or (C) fused with 40% DL, 60% PO 2:1 PE/PG liposomes by multiple freeze-thaw cycles. Data represented as mean ± sem, n = 3 independent samples. P-values are calculated using a two-tailed student's t-test on the *P_1_* photobleaching data, and using the 𝜒^2^ test on the (*P_1_,P_2_,P_3+_*) photobleaching probability distributions, designated in brackets (*, *P* < .05; **, *P* < .01).

| **Sample** | **P_Cy5_** | **incubation time (d)** | **P_1_** | **P_2_** | **P_3+_** | ***P*-value** |
| --- | --- | --- | --- | --- | --- | --- |
| A: 0% DL,  0.2 μg/mg | 0.69 ± 0.01 | 4.7 ± 0.7 | 0.39 ± 0.02 | 0.34 ± 0.02 | 0.27 ± 0.04 |  |
|  |  | 14.3 ± 0.3 | 0.37 ± 0.03 | 0.35 ± 0.01 | 0.27 ± 0.03 |  |
| B: 1:1 dilution of A with 0% DL |  | 4.7 ± 0.7 | 0.44 ± 0.01 | 0.31 ± 0.00 | 0.24 ± 0.01 | ns, *P_AB_* = 0.06  (ns, *P_AB_* = 0.59) |
|  |  | 14.3 ± 0.3 | 0.36 ± 0.08 | 0.34 ± 0.03 | 0.30 ± 0.05 | ns, *P_AB_* = 0.87  (ns, *P_AB_* = 0.80) |
| C: 1:1 dilution of A with 40% DL |  | 4.7 ± 0.7 | 0.52 ± 0.02 | 0.27 ± 0.01 | 0.21 ± 0.01 | **, *P_AC_* = 0.007  *, *P_BC_* = 0.02  (*, *P_AC_* = 0.03  ns, *P_BC_* = 0.28) |
|  |  | 14.3 ± 0.3 | 0.49 ± 0.04 | 0.28 ± 0.02 | 0.23 ± 0.02 | ns, *P_AC_* = 0.07  ns, *P*_BC_ = 0.20  (*, *P_AC_* = 0.05  *, *P_BC_* = 0.03) |
