# Supplementary material for: Membrane transporter dimerization driven by differential lipid solvation energetics of dissociated and associated states
Source: eLife. 2021 Apr 7;10:e63288. doi: 10.7554/eLife.63288 (PMC8116059; doi:10.7554/eLife.63288)
Supplement: Figure 5—source data 1. — Data is represented as mean ± sem, with independent sample preparations, n. Statistical tests were calculated using a two-tailed, unpaired parametric student's t-test on P1 data, and a χ2 test for the mean (P1,P2,P3+) distributions, in brackets (*, p ≤. 05; **, p ≤. 01; ***, p ≤. 001; ****, p ≤. 0001,). ΔG° is calculated for each FDimer value where 0 < FDimer < 1, and ΔΔG = ΔG°(x% DL)-mean(ΔG°(0% DL)), n in brackets. [file elife-63288-fig5-data1.docx]

**Figure 5 - source data 1. Photobleaching titration of CLC-ec1 WT-Cy5 in 2:1 PE/PG mixed DL/PO membranes.** Data is represented as mean ± sem, with independent sample preparations, n. Statistical tests were calculated using a two-tailed, unpaired parametric student's t-test on *P_1_* data, and a 𝜒^2^ test for the mean (*P_1_,P_2_,P_3+_*) distributions, in brackets (*, *P* ≤ .05; **, *P* ≤ .01; ***, *P* ≤ .001; ****, *P* ≤ .0001,). *ΔG°* is calculated for each *F_Dimer_* value where 0 < F_Dimer_ < 1, and *ΔΔG = ΔG°(x% DL)-mean(ΔG°(0% DL))*, n in brackets.

| **% DL (w/w)** | **P_Cy5_** | **incubation time (d)** | **P_1_** | **P_2_** | **P_3+_** | **n** | ***P*-value** | **F_dimer_** | **ΔΔG (kcal/mole)** |
| --- | --- | --- | --- | --- | --- | --- | --- | --- | --- |
| 0 | 0.65 ± 0.01 | 5.1 ± 0.9 | 0.46 ± 0.02 | 0.38 ± 0.01 | 0.17 ± 0.02 | 9 |  | 0.81 ± 0.05 |  |
| 1e-8 | 0.65 ± 0.02 | 4.0 ± 0.6 | 0.50 ± 0.06 | 0.33 ± 0.01 | 0.18 ± 0.05 | 4 | ns, 0.41  (ns, 0.67) | 0.69 ± 0.14 | 0.5 ± 0.9 (4) |
| 1e-7 | 0.64 ± 0.01 | 4.3 ± 0.5 | 0.45 ± 0.05 | 0.34 ± 0.01 | 0.21 ± 0.04 | 4 | ns, 0.92  (ns, 0.54) | 0.80 ± 0.11 | 0.0 ± 0.6 (4) |
| 1e-6 | 0.65 ± 0.02 | 4.0 ± 0.6 | 0.50 ± 0.06 | 0.31 ± 0.02 | 0.19 ± 0.04 | 4 | ns, 0.42  (ns, 0.32) | 0.66 ± 0.14 | 1.6 ± 0.4 (3) |
| 1e-5 | 0.64 ± 0.01 | 4.3 ± 0.5 | 0.51 ± 0.06 | 0.31 ± 0.04 | 0.18 ± 0.04 | 4 | ns, 0.33  (ns, 0.31) | 0.63 ± 0.17 | 1.7 ± 0.5 (3) |
| 1e-4 | 0.65 ± 0.01 | 3.6 ± 0.6 | 0.52 ± 0.04 | 0.31 ± 0.02 | 0.17 ± 0.03 | 5 | ns, 0.17  (ns, 0.28) | 0.63 ± 0.10 | 0.9 ± 0.7 (5) |
| 1e-3 | 0.64 ± 0.01 | 3.8 ± 0.8 | 0.51 ± 0.03 | 0.30 ± 0.02 | 0.19 ± 0.03 | 4 | ns, 0.21  (ns, 0.22) | 0.67 ± 0.07 | 1.0 ± 0.4 (4) |
| 1e-2 | 0.66 ± 0.01 | 3.3 ± 0.6 | 0.52 ± 0.03 | 0.32 ± 0.02 | 0.16 ± 0.01 | 4 | ns, 0.10  (ns, 0.35) | 0.62 ± 0.09 | 1.2 ± 0.5 (4) |
| 0.1 | 0.65 ± 0.00 | 3.8 ± 0.8 | 0.52 ± 0.05 | 0.29 ± 0.02 | 0.18 ± 0.04 | 4 | ns, 0.14  (ns, 0.13) | 0.60 ± 0.11 | 1.4 ± 0.4 (4) |
| 1 | 0.65 ± 0.01 | 3.6 ± 0.6 | 0.57 ± 0.03 | 0.27 ± 0.02 | 0.17 ± 0.02 | 5 | **, 0.0094  (*, 0.036) | 0.51 ± 0.09 | 1.7 ± 0.3 (5) |
| 5 | 0.65 ± 0.01 | 7.5 ± 3.9 | 0.59 ± 0.03 | 0.27 ± 0.02 | 0.14 ± 0.02 | 4 | **, 0.0058  (*, 0.015) | 0.43 ± 0.09 | 2.0 ± 0.3 (4) |
| 10 | 0.65 ± 0.01 | 6.0 ± 2.6 | 0.63 ± 0.07 | 0.24 ± 0.02 | 0.13 ± 0.05 | 3 | **, 0.0062  (***, 0.0007) | 0.32 ± 0.16 | 2.5 ± 0.6 (3) |
| 20 | 0.65 ± 0.01 | 5.0 ± 1.2 | 0.73 ± 0.02 | 0.20 ± 0.01 | 0.07 ± 0.01 | 4 | ****,<0.0001  (****,<0.0001) | 0.07 ± 0.04 | 3.2 ± 0.0 (2) |
| 40 | 0.65 ± 0.01 | 4.5 ± 1.3 | 0.79 ± 0.02 | 0.16 ± 0.01 | 0.05 ± 0.01 | 4 | ****,<0.0001  (****, <0.0001) | 0.00 ± 0.00 | ND |
| 80 | 0.65 ± 0.01 | 4.5 ± 1.3 | 0.88 ± 0.03 | 0.10 ± 0.02 | 0.02 ± 0.01 | 3 | ****,<0.0001  (****,<0.0001) | 0.00 ± 0.00 | ND |
