# Supplementary material for: Membrane transporter dimerization driven by differential lipid solvation energetics of dissociated and associated states
Source: eLife. 2021 Apr 7;10:e63288. doi: 10.7554/eLife.63288 (PMC8116059; doi:10.7554/eLife.63288)
Supplement: Figure 5—source data 2. — CLC-ec1-Cy5 proteoliposomes (0.1 μg/mg) in 100% PO, 2:1 PE/PG were dialyzed alone (-DL) or in the presence of a cassette containing >20% DL (+DL). The p-value was calculated using a χ2 test on the mean (P1,P2,P3+) photobleaching probability distributions. Data is represented as mean ± standard deviation. [file elife-63288-fig5-data2.docx]

**Figure 5 - source data 2. Testing for DL contamination during dialysis.** CLC-ec1-Cy5 proteoliposomes (0.1 μg/mg) in 100% PO, 2:1 PE/PG were dialyzed alone (-DL) or in the presence of a cassette containing > 20% DL (+DL). The *P*-value was calculated using a 𝜒^2^ test on the mean (*P_1_,P_2_,P_3+_*) photobleaching probability distributions. Data is represented as mean ± standard deviation.

| **sample** | **P_Cy5_** | **incubation time (d)** | **P_1_** | **P_2_** | **P_3+_** | **n** | ***P*-value** |
| --- | --- | --- | --- | --- | --- | --- | --- |
| -DL | 0.65 ± 0.02 | 5 ± 3 | 0.46 ± 0.06 | 0.38 ± 0.04 | 0.17 ± 0.05 | 9 |  |
| +DL |  |  | 0.53 ± 0.09 | 0.31 ± 0.02 | 0.20 ± 0.10 | 3 | ns, 0.22 |
