# Supplementary material for: Membrane transporter dimerization driven by differential lipid solvation energetics of dissociated and associated states
Source: eLife. 2021 Apr 7;10:e63288. doi: 10.7554/eLife.63288 (PMC8116059; doi:10.7554/eLife.63288)
Supplement: Figure 5—source data 3. — Lipids are prepared at 20 mg/mL total lipid mass, and the % DL mixtures are prepared by combining stock solutions, w/w of total lipid. Note, the headgroup mole fraction remains nearly constant and ranges from χPE = 0.68–0.69 and χPG = 0.31–0.32 across the range of % DL studied. MWPOPE = 717.996 g/mole, MWPOPG = 770.989 g/mole, MWDLPE = 579.746 g/mole, MWDLPG = 632.739 g/mole. [file elife-63288-fig5-data3.docx]

**Figure 5 - source data 3. DL and PO concentrations, mole fraction and molality in the titrated DL/PO 2:1 POPE/POPG lipid bilayers**. Lipids are prepared at 20 mg/mL total lipid mass, and the % DL mixtures are prepared by combining stock solutions, w/w of total lipid. Note, the headgroup mole fraction remains nearly constant and ranges from 𝜒_PE_ = 0.68-0.69 and 𝜒_PG_ = 0.31-0.32 across the range of % DL studied. MW_POPE_ = 717.996 g/mole, MW_POPG_ = 770.989 g/mole, MW_DLPE_ = 579.746 g/mole, MW_DLPG_ = 632.739 g/mole.

| **mass ratio** | **Concentration** | | | **Lipid mole fraction** | | **Molality** | | **Protein mole fraction** |
| --- | --- | --- | --- | --- | --- | --- | --- | --- |
| **% DL (w/w)** | **[Lipid] (mM)** | **[DL] (mM)** | **[PO] (mM)** | **𝜒_DL_** | **𝜒_PO_** | **m_DL_ (moles/ Kg)** | **m_PO_ (moles/ Kg)** | **𝜒_reconst._**  **(subunits/lipid)** |
| 0 | 27.2 | 0.0 | 27.2 | 0.00 | 1.00 | 0.00 | 1.36 | 1.0e-6 |
| 1e-8 | 27.2 | 3.4e-9 | 27.2 | 1.2e-10 | 1.00 | 1.7e-10 | 1.36 | 1.0e-6 |
| 1e-7 | 27.2 | 3.4e-8 | 27.2 | 1.2e-09 | 1.00 | 1.7e-9 | 1.36 | 1.0e-6 |
| 1e-6 | 27.2 | 3.4e-7 | 27.2 | 1.2e-08 | 1.00 | 1.7e-8 | 1.36 | 1.0e-6 |
| 1e-5 | 27.2 | 3.4e-6 | 27.2 | 1.2e-07 | 1.00 | 1.7e-7 | 1.36 | 1.0e-6 |
| 1e-4 | 27.2 | 3.4e-5 | 27.2 | 1.2e-06 | 1.00 | 1.7e-6 | 1.36 | 1.0e-6 |
| 1e-3 | 27.2 | 3.4e-4 | 27.2 | 1.2e-05 | 1.00 | 1.7e-5 | 1.36 | 1.0e-6 |
| 1e-2 | 27.2 | 3.4e-3 | 27.2 | 1.2e-04 | 1.00 | 1.7e-4 | 1.36 | 1.0e-6 |
| 0.1 | 27.2 | 3.4e-2 | 27.2 | 0.01 | 0.99 | 1.7e-3 | 1.36 | 1.0e-6 |
| 1 | 27.3 | 3.4e-1 | 26.9 | 0.01 | 0.99 | 1.7e-2 | 1.35 | 1.0e-7 |
| 5 | 27.5 | 1.7 | 25.9 | 0.06 | 0.94 | 8.4e-2 | 1.29 | 9.9e-7 |
| 10 | 27.8 | 3.4 | 24.5 | 0.12 | 0.88 | 0.17 | 1.22 | 9.8e-7 |
| 15 | 28.2 | 5.0 | 23.1 | 0.18 | 0.82 | 0.25 | 1.16 | 9.7e-7 |
| 20 | 28.5 | 6.7 | 21.8 | 0.24 | 0.77 | 0.34 | 1.09 | 9.6e-7 |
| 30 | 29.1 | 10.1 | 19.1 | 0.35 | 0.65 | 0.50 | 0.95 | 9.4e-7 |
| 40 | 29.7 | 13.4 | 16.3 | 0.45 | 0.55 | 0.67 | 0.82 | 9.2e-7 |
| 50 | 30.4 | 16.8 | 13.6 | 0.55 | 0.45 | 0.84 | 0.68 | 9.0e-7 |
| 70 | 31.6 | 23.5 | 8.2 | 0.74 | 0.26 | 1.17 | 0.41 | 8.6e-7 |
| 80 | 32.3 | 26.8 | 5.4 | 0.83 | 0.17 | 1.34 | 0.27 | 8.4e-7 |
| 100 | 33.5 | 33.5 | 0.0 | 1.00 | 0.00 | 1.68 | 0.00 | 8.1e-7 |
